# Supplementary figures and images for: Associations of serum DNA methylation levels of chemokine signaling pathway genes with mild cognitive impairment (MCI) and Alzheimer’s disease (AD)
Source: PLoS One. 2023 Dec 1;18(12):e0295320. doi: 10.1371/journal.pone.0295320 (PMC10691689; doi:10.1371/journal.pone.0295320)

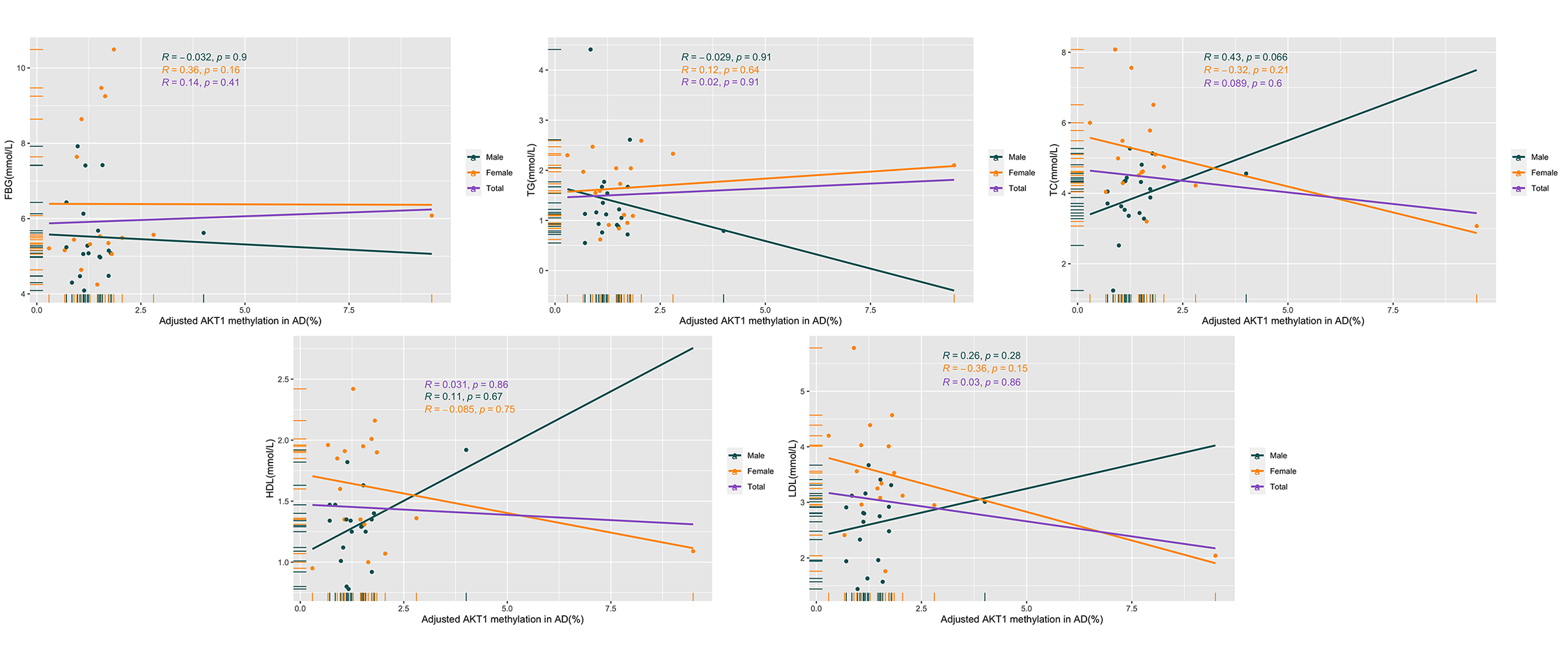

Supplement: S1 Fig — (TIF) [file pone.0295320.s001.tif]

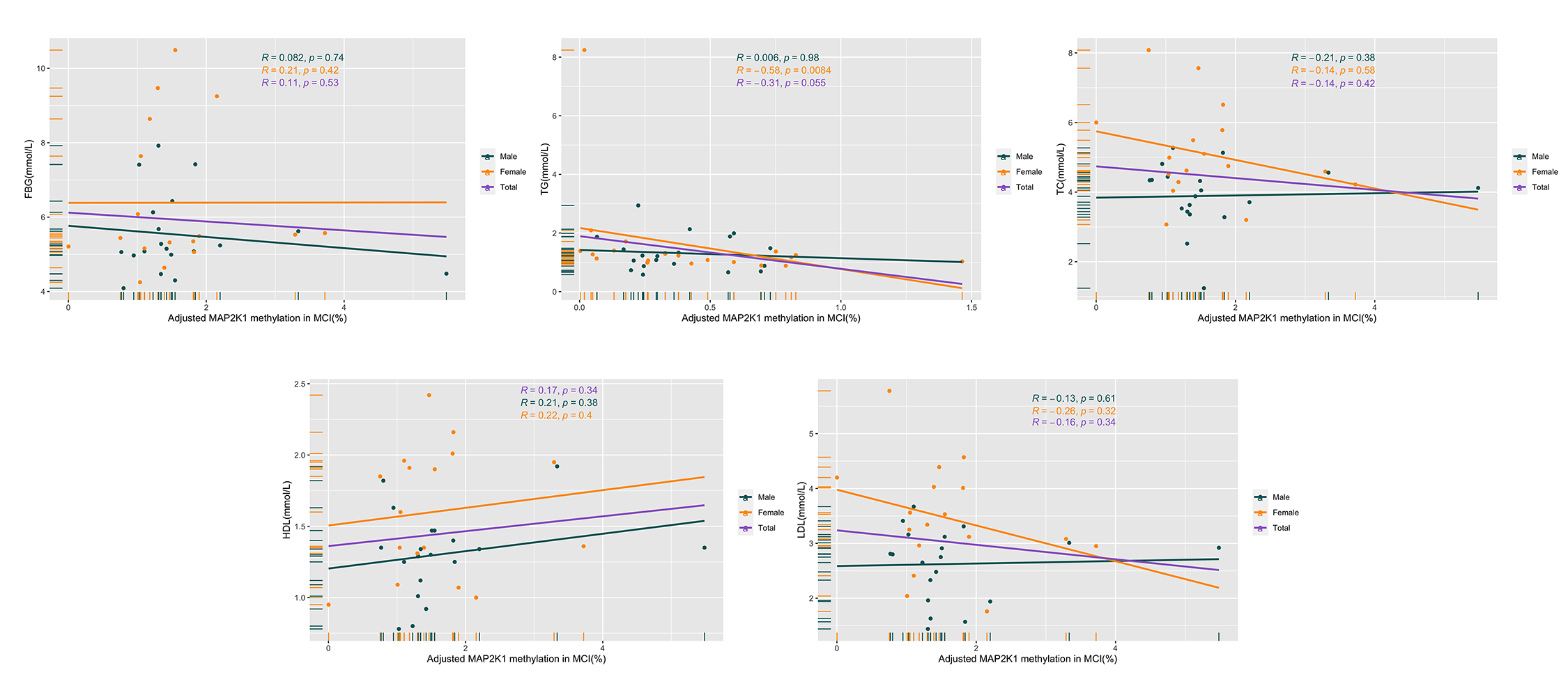

Supplement: S2 Fig — (TIF) [file pone.0295320.s002.tif]
