# Supplementary material for: Associations of serum DNA methylation levels of chemokine signaling pathway genes with mild cognitive impairment (MCI) and Alzheimer’s disease (AD)
Source: PLoS One. 2023 Dec 1;18(12):e0295320. doi: 10.1371/journal.pone.0295320 (PMC10691689; doi:10.1371/journal.pone.0295320)
Supplement: S1 Table — (DOCX) [file pone.0295320.s003.docx]

**S1 Table. Primer sequences for qMSP and single nucleotide polymorphism analysis of *APOE*.**

| **Gene** | **Forward Primer (5' - 3')** | **Reverse Primer (5' - 3')** | **Ta** |
| --- | --- | --- | --- |
| *CXCL5* | ATATAGTAGCGGTAGGATTAGT | TCCCAACCTCTACGAAAT | 58℃ |
| *ADCY2* | CGGCGAGGATTACGATTA | GCTCAACTTCAATCACACTT | 60℃ |
| *HCK* | GTATTGTTGCGGATTGTT | AAACCTAAACGACCCAAA | 58℃ |
| *MAP2K1* | TTATCGTAGAGGTGGAGGT | GAAACAAACAAATAAACCCAACT | 60℃ |
| *AKT1* | GGGCGGTTAAGAGTGATT | ACCTCCTATCCTAACATCCT | 60℃ |
| *WASL* | GTGAAGCGAGAGTTAGAA | AACCTATCCTCCAACCTA | 58℃ |
| *RAP1B* | TTTATTTGTTGCGGGAAGT | TCTCTACACGATAACTACTACC | 60℃ |
| *APOE* rs7412 rs429358 | ATCTGTCTCTGTCTCCTTCT | CTCGAACCAGCTCTTGAG | 58℃ |
