# Supplementary material for: Associations of serum DNA methylation levels of chemokine signaling pathway genes with mild cognitive impairment (MCI) and Alzheimer’s disease (AD)
Source: PLoS One. 2023 Dec 1;18(12):e0295320. doi: 10.1371/journal.pone.0295320 (PMC10691689; doi:10.1371/journal.pone.0295320)
Supplement: Attachment — Submitted filename: Response to reviewers.docx [file pone.0295320.s005.docx]

Dear Reviewer：

Thank you for your letter and for the comments concerning our manuscript entitled “Associations of serum DNA methylation levels of chemokine signaling pathway genes with mild cognitive impairment (MCI) and Alzheimer's disease (AD)”. (ID: PONE-D-23-21380). Those comments are all valuable and very helpful for revising and improving our paper, as well as the important guiding significance to our researches. We have studied comments carefully and have made corrections which we hope meet with approval. Revised portions are marked with highlights in the paper. The main corrections in the paper and the responds to the reviewer’s comments are as following：

Responds to the reviewer's comments:

Reviewer #1:

1. Response to comment：In the introduction, the authors did not summarize the previous research precisely. The authors should mention previous studies and describe the significance of the subject of this study clearly.

Response: We think this is an excellent suggestion. We have already added this section with highlights in the fourth and fifth paragraphs in introduction according to the Reviewer’s suggestion.

2. Response to comment：The authors determined the DNA methylation levels of the seven genes; however, their logic and bases were unclear. The authors should describe it clearly. In addition, did these protein levels change in the blood of MCI and AD patients? Were these changes related to the pathogenesis of these diseases? What’s the specificity?

Response: Thanks for this good suggestion. We have added the logical relationship content about the seven genes with highlight at the end of the second paragraph in discussion. About whether protein levels have changed in the blood of MCI and AD patients, due to the impact of blood sample storage and logistics during the COVID-19 pandemic, this study could not further determine the protein levels of *AKT1* and *MAP2K1* genes in blood, so it could not fully explore their role and mechanism in the pathogenesis of AD or MCI, which is the limitation of this study. However, in our recent follow-up study, we have begun to perform function experiments in vitro cell on the above genes, and intend to analyze the protein levels of these genes in blood, in order to discover the possible mechanism by these genes cause AD or MCI.

3. Response to comment：The authors identified significant differences of *ADCY2*, *MAP2K1* and *AKT1*’s DNA methylation levels. Were these levels related to the seriousness or score of MCI and AD patients? How about the duration of these diseases?

Response: The blood samples in this study came from epidemiological survey. Due to the limited conditions and funds in epidemiological survey, only MMSE and MoCA scales were scored in the survey, and the disease course of each MCI and AD patient could not be recorded in detail, nor could MRI examination of the head or hippocampus of all patients be conducted. Therefore, the score was not analyzed for disease duration in this study. Since the judgment of the severity of AD or MCI disease needs to be combined with the score and the MRI results of the head or hippocampus, in order to prevent bias, the correlation between the DNA methylation levels of *ADCY2*, *MAP2K1* and *AKT1* and the severity of the disease is not analyzed in this study. However, this is a very good suggestion, and in our follow-up study, objective examination results such as disease course and MRI of the head or hippocampus were included in the collection for deeper analysis.

4. In the title of this manuscript, “chemokine signaling pathway” was described. Were chemokines changed in the MCI and AD patients of the present study? Was the change in *ADCY2*, *MAP2K1*, and *AKT1*’s DNA methylation levels related to these cytokines?

Response: Thanks to this suggestion, due to the impact of specimen storage and logistics during the COVID-19 pandemic, the detection reagents could not be in place in time, so the chemokines could not be determined in this study. In our follow-up study, we have included this in the research scope for analysis.
